# Endogenous Endophthalmitis: yield of the diagnostic evaluation

**DOI:** 10.1186/s12886-020-01418-9

**Published:** 2020-04-07

**Authors:** Kathleen A. Regan, Nila S. Radhakrishnan, Jon D. Hammer, Benjamin D. Wilson, Lara Beth Gadkowski, Siva S. R. Iyer

**Affiliations:** 1 Department of Ophthalmology and Visual Sciences, University of Wisconsin School of Medicine and Public Health, Madison, WI USA; 2 Department of Medicine, Division of Hospital Medicine, University of Florida College of Medicine, Gainesville, FL USA; 3 Department of Ophthalmology, University of Florida College of Medicine, 1600 SW Archer Rd, Gainesville, FL 53610 USA; 4 Department of Ophthalmology, State University of New York Downstate Medical Center, Brooklyn, NY USA; 5 Department of Medicine, Division of Infectious Diseases & Global Medicine, University of Florida College of Medicine, Gainesville, FL USA

**Keywords:** Endogenous endophthalmitis, Intravenous drug abuse, Sepsis, Diagnostic imaging, Hospital discharge

## Abstract

**Background:**

Endogenous endophthalmitis is an infection of the eye secondary to sepsis, occurring in 0.04–0.5% of bacteremia or fungemia. Risk factors include intravenous drug abuse (IVDA), diabetes, indwelling catheters, and immune suppression. Many patients have known or suspected bacteremia or fungemia; however, culture yield is reported to be low (approximately 50%). The purpose of this study is to elucidate the yield of diagnostic evaluation including microbial cultures over a 6.5 year period at an academic center in the United States.

**Methods:**

Retrospective chart review of patients with endogenous endophthalmitis at the University of Florida from June 2011 to February 2018.

**Results:**

Included are 40 eyes of 35 patients. Endophthalmitis was secondary to an endogenous source in 23.5% of all endophthalmitis cases observed. Intraocular culture positivity was 28.6% overall but was 0% after initiation of systemic antibiotics. Most commonly identified organisms from the eye were coagulase-negative *Staphylococcus* and *Candida*. Blood culture positivity was 48.6%, most commonly *Staphylococcus*. IVDA was noted with increasing frequency as a risk factor. Diagnosis of endophthalmitis upon hospital admission was associated with a higher intraocular culture positivity (*P* = 0.040) and a shorter hospital stay (*P* = 0.035). Computed tomography (CT) and magnetic resonance imaging (MRI) were the highest yield imaging modalities; X-ray and non-ocular ultrasound were less diagnostically useful. Echocardiogram was positive by transesophageal route (TEE) in 22% and in 9% by transthoracic (TTE) testing. Following discharge from the hospital, 48.4% of patients failed to follow up with outpatient ophthalmology.

**Conclusions:**

Based on the results of this study, the interdisciplinary team should consider directed imaging, eye cultures prior to antimicrobial administration, thorough history for IVDA, and caution with premature discharge from the hospital.

## Background

Endophthalmitis is a rare but devastating infection of ocular tissues that may result secondary to eye trauma, surgery, or systemic infection. Treatment consists of intravitreal antibiotics. Intraocular culture may be obtained by needle aspirate at the bedside or by surgical vitrectomy.

Endogenous endophthalmitis is a rare complication of sepsis, found in less than 0.5% of patients with fungemia and 0.04% of patients with bacteremia [1]. Endogenous endophthalmitis develops when pathogens cross the blood-ocular barrier, resist the body’s immune system, and proliferate within the eye [2]. The blood-ocular barrier is thought to be strong in most cases, as only few pathogens in the vitreous cavity can lead to endophthalmitis [3]. Presenting symptoms include eye pain and blurry vision; hypopyon, a collection of white blood cells in the anterior chamber, may be present on exam [2]. Risk factors for endogenous endophthalmitis include human immunodeficiency virus (HIV) infection, endocarditis, meningitis, lymphoma or leukemia, and abscess of organ or joint [1]. Longer hospital stays and more intensive care required are also associated with an increased risk [1]. The most common cause of bacterial endogenous endophthalmitis in the West is Gram-positive infection [1, 4]. Gram-negative infection, especially *Klebsiella pneumoniae*, are seen more commonly in Asia [2, 5–7]. Candida is the most common fungal cause of endogenous endophthalmitis [1].

This study’s purpose is to analyze the yield of diagnostic evaluation, and better understand culture characteristics of endogenous endophthalmitis cases that presented over the course of 6.5 years to a southern tertiary care academic center in the United States.

## Methods

Institutional review board (IRB) approval was obtained at the University of Florida. Procedures followed were in accordance with the ethical standards of the IRB and with the Helsinki Declaration (1964, amended most recently in 2008) of the World Medical Association.

This study was a retrospective, noncomparative case series from June 2011 to February 2018. Electronic medical records were searched for a diagnosis code of endophthalmitis and verified by a single reviewer to ensure correct coding. Relevant data, including treatments, results of diagnostic testing, and vision, were recorded. Patients were included if there was a clinical diagnosis of endogenous endophthalmitis and excluded if there was a known or suspected exogenous source. Patients were included whether they were pre-existing inpatients or new patients to the hospital. The date of diagnosis of endophthalmitis was recorded in relation to the admission date. Statistical analysis was performed with two-tailed t-tests and Chi square. Summary statistics were reported as mean ± standard deviation. Snellen vision was converted to the logarithm of the minimum angle of resolution (logMAR) for the purposes of statistical analysis. Non-numeric vision was converted to logMAR as follows [8]: count fingers 2.0, hand motions 2.3, light perception 2.7, no light perception 3.0.

## Results

### Patient population and risk factors

Over the approximately 6.5 years included in this study, 149 patients were diagnosed with endophthalmitis at our institution. Of these, 35 patients (23.5%), including 5 bilateral, were secondary to an endogenous source. The mean age of these 35 patients was 55 ± 18 years old.

The most common identifiable source of infection was intravenous drug abuse (IVDA), observed in 8 patients (22.9%; Table 1). The number of cases with this associated risk factor was noted to increase over the course of the study, with 0 patients in 2011, 2012, and 2013, 1 in 2014 and 2015, 2 in 2016, and 4 in 2017. The most common comorbidity observed in this population was diabetes, reported in 16 patients (45.7%; Table 2).

**Table 1:** Source of Infection

*Cause*	*n*	*%*
unknown	11	31.4
IVDA	8	22.9
port/catheter	5	14.3
extremity (shoulder, leg)	4	11.4
GI (liver, colon)	2	5.7
endocarditis	2	5.7
pneumonia	1	2.9
dermatitis	1	2.9
UTI	1	2.9
total:	35	

*IVDA* intravenous drug abuse, *GI* gastrointestinal, *UTI* urinary tract infection

**Table 2:** Associated Co-Morbidities

*Comorbidities*	*n*	*%*
DM	16	45.7
leukemia/lymphoma	7	20.0
heart disease (CAD, CHF)	6	17.1
lung disease (COPD, ILD)	5	14.3
solid cancer	4	11.4
ESRD	4	11.4
GI ulcer	2	5.7
heart valve replacement	1	2.9

*DM* diabetes mellitus, *IVDA* intravenous drug abuse, *CAD* coronary artery disease, *CHF* congestive heart failure, *COPD* chronic obstructive pulmonary disease, *ILD* interstitial lung disease, *ESRD* end-stage renal disease, *GI* gastrointestinal

### Diagnostic evaluation

Most patients were admitted as inpatients; however, four patients were treated as outpatients only. In most cases, Infectious Diseases (ID) was consulted for evaluation and treatment recommendations; in four patients, ID was not consulted (including 3 of the outpatient cases). The average inpatient stay was 16.1 ± 11.9 days. In 15 eyes (37.5%), the diagnosis of endophthalmitis was present on admission. In the remaining cases, the duration from admission to diagnosis was 5.7 ± 5.1 days. Of the 20 eyes that were diagnosed with endophthalmitis on or within 1 day after admission, vision decline was a primary complaint by the patient. 16/20 had count fingers vision or worse. For the remaining half, endophthalmitis was a secondary diagnosis. The diagnosis of endophthalmitis prior to admission was associated with a shorter hospital stay (11.4 ± 8.5 days) compared with the diagnosis made after admission (20.25 ± 13.2 days; *P* = 0.035). Outside of the 20 patients that were diagnosed either at or within a day of admission, who had primary vision complaints, other non-ocular reasons for admission ranged from shortness of breath to septic shock.

Our study found that treating physicians performed diagnostic evaluations at their discretion without a common pathway in place. The four patients managed as outpatients did not have any diagnostic imaging obtained. Seven patients did not have imaging obtained as part of their inpatient stay, the remaining 24 patients had x-rays (100%), computed tomography (CT; 87.5%), magnetic resonance imaging (MRI; 25%), or ultrasound (54.2%) during their inpatient stay (Table 3). Percent yield of relevant findings that suggest the source of infection were calculated for each imaging type. Of the 24 patients who had x-rays during their admission, 4.4 ± 5.5 x-rays were obtained per patient, with relevant findings in 2.7%. Of the 21 patients who had CT testing, 1.6 ± 1 were obtained per patient, with a yield of 58.3%. Of the six patients with MRI testing, 1.3 ± 0.5 MRIs were obtained per patient, with a positivity of 58.3%. Of the 12 patients with ultrasound testing, 1.4 ± 1 studies were obtained per patient, with a yield of 8.3%. Ocular ultrasound was not specifically evaluated in our study as this practice was not consistent among the many providers over the course of the years reviewed.

**Table 3:** Diagnostic Imaging

X-ray	# of patients	MRI	# of patients
No x-rays	11	No MRIs	29
All x-rays negative	19	All MRIs negative	1
Pneumonia	2	Vasculitis (brain)	2
Dental abscess/mucositis	2	Chorioretinitis	1
Liver abscess	1	Septic arthritis	1
		Orbital infection	1
**CT (may have multiple positive findings per scan)**		**Ultrasound**	
No CT scans	14	No ultrasounds	22
All CT scans negative	8	All ultrasounds negative	11
Endophthalmitis	4	Pancreatic abscess	1
Pneumonia	5		
Pyelonephritis	2		
Pancreatitis	1		
Intravitreal gas	1		
Preseptal cellulitis	1		
Septic arthritis	1		
Liver abscess	1		
Splenic/renal septic infarct	1		
Colitis	1		
Dental abscess	1		
Myositis	1		

Transthoracic echocardiogram (TTE) was obtained in 26 patients, with 92.3% negative findings. Positive findings were aortic regurgitation in one patient and pericardial effusion in another. Transesophageal echocardiogram (TEE) was then obtained in 18 patients, 4 of which revealed vegetations (22%). Two lumbar punctures were obtained, both with negative cultures.

In 35 eyes, intraocular cultures were obtained, with 10 positive samples (28.6%). If anti-infectious agents were initiated prior to intraocular culture, no culture returned as positive. With the patients who received prior antibiotics excluded, 41.7% had positive intraocular fluid cultures. When the diagnosis of endophthalmitis was made on the day of admission, the culture positivity rate was 46.7%, higher than the positivity rate when diagnosed after admission (15%; *P* = 0.040). Intraocular culture positivity was not significantly associated with hospital stay (12.4 ± 6.5 days for positive culture, 18.8 ± 13.8 days for negative culture; *P* = 0.11). The most common bacteria identified was coagulase-negative *Staphylococcus* (CoNS), while the most common yeast was *Candida* species; these pathogens were equally common (*n* = 3; Table 4).

**Table 4:** Culture Results

*Ocular*	*# eyes*		*Blood*	*# patients*
*Candida* (all spp)	3		MRSA	7
Coagulase-negative *Staphylococcus*	3		Coagulase-negative *Staphylococcus*	5
*Micrococcus*	2		*Candida* (all spp)	3
MRSA	1		MSSA	2
*E coli*	1		*S. viridans*	1
*Klebsiella pneumoniae*	1		*E coli*	1
*Bacillus*	1		*Klebsiella pneumoniae*	1
Polymicrobial	1		Polymicrobial	1
*Other Sources*	*# patients*	*Positive*	*% yield*	
skin	1	1 (*Candida*)	100%	
abscess/septic joint	5	4 (3 MRSA, 1 *E. coli*)	80%	
stool	2	1 (*C. difficile*)	50%	
sputum	5	1 (*Klebsiella*)	20%	
urine	15	2 (1 *Candida*, 1 polymicrobial: *Candida + Klebsiella*)	13.3%	
IV catheter	2	0	0%	
heart valve	1	0	0%	

Blood cultures were obtained in 33 patients, with 16 positive samples (48.5%). Methicillin-resistant *Staphylococcus aureus* (MRSA) was most common organism identified (Table 4). No difference in blood culture positivity was seen when diagnosis of endophthalmitis was made before or after admission (*P* = 0.58), and blood culture positivity was not significantly associated with length of inpatient hospital stay (*P* = 0.3) or duration of antibiotics (41.3 ± 19.3 days positive vs. 30.5 ± 16.2 days negative cultures; *P* = 0.1).

Urine culture was obtained in 15 patients, with 2 positive (13.3%). Further cultures were obtained in 11 patients; skin and abscess cultures had the highest yields (Table 4).

### Treatment

Most eyes (85%) were treated with a sample of intraocular fluid by needle aspirate, intravitreal injection of antibiotics, or both. Eight of these were treated with vitrectomy surgery later. Five patients were treated primarily with vitrectomy, two of these requiring a second delayed vitrectomy. The decision for vitrectomy was made by the treating vitreoretinal surgeon. There was no documented visual acuity indication for vitrectomy.

Eyes treated with primary vitrectomy had an intraocular culture positivity of 60%; when two eyes from the same patient who had previously received systemic antibiotics were excluded, the culture positivity rate was 100%. Patients treated with vitrectomy at any time did not have a significant difference in vision outcome from patients who were never treated with vitrectomy (*P* = 0.2). One patient was treated primarily with enucleation, and a second was treated with delayed enucleation. Enucleations were due to severity of infections, and painful blind eyes.

Intravitreal treatment regimen was most commonly vancomycin and ceftazidime together and/or in combination with a different agent (voriconazole, amphotericin, clindamycin). Multiple injections were administered in 17 eyes, with an average of 1.8 ± 1.4 injections per patient. A variety of systemic antibiotics and antifungals were chosen during treatment. Mean number of anti-infectious agents was 3.3 ± 1.8. Systemic antibiotics were administered prior to the diagnosis of endophthalmitis in 16 eyes (40%). Vancomycin was the most commonly administered systemic agent, given in 82.9% of patients. Systemic agents were administered for an average 34.1 ± 18.7 days. Duration of systemic antibiotics was not associated with intraocular culture positivity (*P* = 0.44). In 32 eyes (80%), topical antibiotics were administered, moxifloxacin being the most common (42.5%).

### Outcomes

Four patients died since last follow up. One died of septic shock during the admission when endophthalmitis was diagnosed. One was discharged to hospice. One died of an arrhythmia from hyperkalemia during hemodialysis, temporally unrelated to the endophthalmitis, though the dialysis access port was considered the source of the infection. One died of unknown causes outside of our hospital system.

Average vision at presentation was logMAR 1.9 ± 0.9. Of the 31 patients treated inpatient for their endophthalmitis, 15 (48.4%) never followed up outpatient with our ophthalmology service. Twenty patients (including four who were never admitted) were followed by outpatient ophthalmology after the diagnosis of endophthalmitis for an average of 258 ± 418 days. Average vision for this subset was logMAR 1.2 ± 1.1 at last follow up, significantly improved from presentation (logMAR 1.9 ± 0.8; *P* = 0.007).

## Discussion

### Patient population

In the literature, rates of endogenous endophthalmitis among all causes are reported as 7.7–13.2% [9–11]. At our center, endogenous endophthalmitis accounted for 23.5% of all endophthalmitis cases. We cannot definitively explain the higher rate seen at our center, but we attribute the large tertiary care hospital receiving many referrals throughout the region to this finding, which may reflect selection bias.

As seen in our study, IVDA is a common source of entry for pathogens into the bloodstream, accounting for 25 to 53% of endogenous endophthalmitis at some centers [12]. IVDA-associated endophthalmitis was noted with increasing frequency over the course of this study, likely correlating to the rise of IVDA in the community [13]. Common to our study, diabetes and cancer have been identified as common comorbid factors in other studies [2, 14]. In Asian countries, liver abscess is more commonly reported as the source of infection [6, 10].

We, like other studies [1], consider length of inpatient admission as a marker of the severity of infection or systemic health, as patients with a well-controlled systemic infection may be discharged to complete an antibiotic course as an outpatient. In this study, the length of systemic antibiotics (34.1 ± 18.7 days) was double the length of the average inpatient admission (16.1 ± 11.9 days). In our series, diagnosis of endophthalmitis on admission was associated with a shorter hospital stay, perhaps because appropriate treatment was able to be initiated more rapidly or because of less complex medical management required.

### Diagnostic testing

Intraocular culture positivity was overall 28.6%, but 41.7% when systemic anti-infective agents had not yet been administered. Likewise, when endophthalmitis was diagnosed on admission, the culture positivity rate was higher than when diagnosis was made later during hospital admission. Given the low rate of culture positivity (0%) when anti-microbial drugs had already been administered, ophthalmologists may wish to defer obtaining a vitreous aspirate, as the risk of may outweigh the benefits of identification of the infectious pathogen when the yield is poor.

Intraocular culture positivity is often low in endogenous endophthalmitis cases, reported at 14–43% in the literature of other centers [2, 4, 6, 15–17]. Given this low diagnostic yield, endophthalmitis must often be treated empirically, such as the initiation of antifungals based on a typical fundus appearance [15]. Primary vitrectomy or polymerase chain reaction (PCR) of the vitreous aspirate may be used to increase diagnostic yield [18].

Blood culture positivity was 48.5%, similar to what has been reported elsewhere [4, 16]. As in other studies from Western centers [4], Gram-positive organisms were the most commonly identified pathogen. In some centers, fungus is frequently identified in endogenous endophthalmitis [14, 19], accounting for approximately half of positive cultures, *Candida* being the most common pathogen [18, 20]. Fungal infections may have better prognosis than bacterial [18, 21]; however, this outcome is not consistent across the literature [22]. *Candida* may be associated with IVDA [20]. Gram-negative organisms were less commonly identified in this series but are more prevalent in East Asian studies and may confer a worse prognosis [6].

In this series, CT and MRI had the highest diagnostic yield for the source or other sites of end-organ infection. Positive findings analysis of CT frequently showed pneumonia (36.8%), septic inflammation of a space/joint (31.6%), and eye or orbit findings (21%). MRI positive findings frequently found tissue inflammation. These included CNS vasculitis or chorioretinitis,(60%), and septic joint inflammation (40%).

Using these studies in a targeted fashion may thus be the preferred modality for imaging evaluation. X-ray and ultrasound had a poor yield. In some cases however, multiple images of the same modality were obtained for correct implant placement (e.g. peripherally inserted central catheter (PICC) line) or for monitoring of treatment effectiveness (e.g. pleural effusions). The acquisition of multiple images particularly in the case of X-rays may reasonably change the interpretation of diagnostic yield as the radiology read may have been influenced by the intent of the imaging study.

### Treatment

While the utility of intravitreal antibiotics in the management of endogenous endophthalmitis is controversial [2], most (95%) of the patients in this study received them, with multiple injections given in 52%. Vancomycin and ceftazidime were the most common intraocular antibiotics given in this study, consistent with the literature conclusions based on intraocular safety and efficacy of the most common agents [2].

No clear consensus exists on indication and timing for vitrectomy in the management of endogenous endophthalmitis. Vitrectomy may be protective to vision and preservation of the eye [2, 20]; however, no significant difference in vision outcomes was seen in this study, and our rate of enucleation was 5% (*n* = 2). Vitrectomy may increase diagnostic culture yield, especially if performed prior to systemic antibiotics; however, in our population, as in others in the literature [12], the sample size is too small to draw strong conclusions. Systemic disease and risks of anesthesia may prevent surgeons from pursuing vitrectomy.

### Outcomes

Two patients in this series were treated with enucleation, accounting for 5% of the total eyes included, compared with 25% in a review [2] from 1976 to 2003. More recent studies [4, 14, 20] report enucleation rate at 8–12%. In this study, however, the poor follow up may limit conclusion regarding globe preservation, as this study included five eyes with no light perception at last exam but never followed up with our outpatient ophthalmology service and may have been enucleated elsewhere. Given the low rate of follow up seen in our series, we caution against premature discharge when the ocular infection is still poorly controlled.

### Limitations

As with all retrospective case series, this study limited by the inability to determine causation and prevent bias. This study is also limited by sample size; however, the number of eyes (40) included in this study is typical of similar single-centered reports in the literature [18, 20]. Our analysis of patient outcomes, especially regarding vision, is limited by the poor follow up to the ophthalmology clinic. Although social issues, re-location, or improvement in condition are all reasons for loss to follow up, we could not identify the specific reasons in our study.

## Conclusions

In this series of 40 eyes with endogenous endophthalmitis, IVDA was noted with increasing frequency as a risk factor for infection. Diagnosis of endophthalmitis upon hospital admission was associated with a higher intraocular culture positivity and a shorter hospital stay. Yield of intraocular culture after initiation of systemic antibiotics was 0%. CT and MRI were the highest yield imaging modalities for diagnostic evaluation. Outpatient follow up to ophthalmology was poor; care should be taken when discharging these patients until they are stable in both their systemic and ocular health. Based on the results of this study, the interdisciplinary team should consider targeted imaging, eye cultures prior to antibiotic administration, thorough history for IVDA, and caution with premature discharge from the hospital.

## Data Availability

The datasets used and/or analyzed during the current study are available from the corresponding author on reasonable request.

## Abbreviations

CoNS: Coagulase-negative *Staphylococcus*
CT: Computed tomography
HIV: Human immunodeficiency virus
ID: INFECTIOUS Diseases
IRB: Institutional review board
IVDA: Intravenous drug abuse
logMAR: Logarithm of the minimum angle of resolution
MRI: Magnetic resonance imaging
MRSA: Methicillin-resistant *Staphylococcus aureus*
PCR: Polymerase chain reaction
TEE: Transesophageal echocardiogram
TTE: Transthoracic echocardiogram
